# The Impact of ACE2 Polymorphisms on COVID-19 Disease: Susceptibility, Severity, and Therapy

**DOI:** 10.3389/fcimb.2021.753721

**Published:** 2021-10-22

**Authors:** Fei Chen, Yankun Zhang, Xiaoyun Li, Wen Li, Xuan Liu, Xinyu Xue

**Affiliations:** Department of Physiology, Jining Medical University, Jining, China

**Keywords:** COVID-19, SARS-CoV-2, ACE2, variants, gene polymorphism, susceptibility, hrsACE2

## Abstract

The coronavirus disease 2019 (COVID-19), caused by severe acute respiratory syndrome coronavirus 2 (SARS-CoV-2), has currently spread worldwide, leading to high morbidity and mortality. As the putative receptor of SARS-CoV-2, angiotensin-converting enzyme 2 (ACE2) is widely distributed in various tissues and organs of the human body. Simultaneously, ACE2 acts as the physiological counterbalance of ACE providing homeostatic regulation of circulating angiotensin II levels. Given that some ACE2 variants are known to cause an increase in the ligand-receptor affinity, their roles in acquisition, progression and severity of COVID-19 disease have aroused widespread concerns. Therefore, we summarized the latest literature and explored how ACE2 variants and epigenetic factors influence an individual’s susceptibility to SARS-CoV-2 infection and disease outcome in aspects of ethnicity, gender and age. Meanwhile, the possible mechanisms for these phenomena were discussed. Notably, recombinant human ACE2 and ACE2-derived peptides may have special benefits for combating SARS-CoV-2 variants and further studies are warranted to confirm their effects in later stages of the disease process. As the uncertainty regarding the severity and transmissibility of disease rises, a more in-depth understanding of the host genetics and functional characteristics of ACE2 variants will not only help explain individual clinical differences of the disease, but also contribute to providing effective measures to develop solutions and manage future outbreaks of SARS-CoV-2.

## Introduction

**Figure 1:**
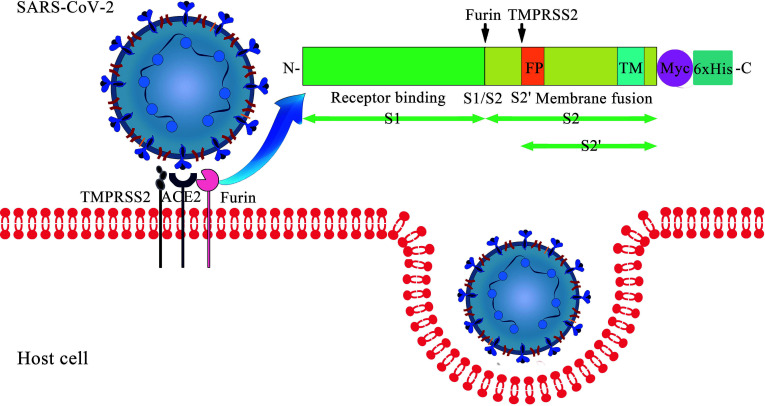
Cell entry of SARS-CoV-2: the interaction between S protein and membrane ACE2.

Coronavirus disease 2019 (COVID-19) poses a tremendous threat to human health. As of 4 August 2021, the infection has its presence around the globe with over 199 million of the world population being infected and has claimed over 4 million lives and counting (WHO, 2021). Severe acute respiratory syndrome coronavirus 2 (SARS-CoV-2), the virus causing COVID-19, is closely related to SARS-CoV with above 85% identity (Gralinski and Menachery, 2020). Similar to SARS-CoV, the entry of SARS-CoV-2 into the host cell is mediated by the surface-anchored spike (S) glycoprotein (**Figure 1**). The protein has two functional units known as “S1” and “S2”, which are responsible for receptor binding and membrane fusion respectively. The receptor-binding domain (RBD) in the S1 binds to the extracellular peptidase domain (PD) of angiotensin-converting enzyme 2 (ACE2), which leads to the exposure of S1/S2 interdomain protease site. Then, the S protein is cleaved by the proprotein convertase furin at the S1/S2 site and the transmembrane serine protease 2 (TMPRSS2) at the S2 site (Bestle et al., 2020). These processes facilitate large-scale conformational changes, triggering the fusion reaction by insertion of the fusion peptide of S2 into the host membrane (Suryamohan et al., 2021). The viral RNA is released for replication and translation upon the internalization of the SARS-CoV-2. Subsequently, new viruses are assembled and released from the cell to start another infection cycle.

As a functional receptor of SARS-CoV-2, full-length ACE2 is a 120-KDa typical zinc-metalloproteinase type I transmembrane protein, which consists of an N-terminal signal peptide of 17 amino acid residues, a PD with its HEXXH + E zinc-binding consensus sequence and a C-terminal Collectrin-like domain (Devaux et al., 2020a). It has two states, i.e. open and closed. In the open state, ACE2 opens widely from its active site, waiting for a ligand to enter. Once the ligand enters ACE2 active site, it will trigger ACE2 to close the active slot (Guo et al., 2020). ACE2 is expressed in essentially all tissues, with the highest expression in the small intestine, kidneys, testis, thyroid, heart, and adipose tissue followed by liver, lungs, colon, bladder, and adrenal gland (Li et al., 2020). Besides, there is another functional form of ACE2 with 555 amino acids that circulates in small amounts in the blood, named soluble ACE2 (sACE2), which is obtained by shedding of the full-length ACE2 through metalloproteinase 17 (ADAM17). It is worth emphasizing that binding of sACE2 to SARS-CoV-2 blocks the entry of the virus into the target cells and consequently, prevents the progression of COVID-19. Up to now, three major ACE2 functions have been described. In addition to being the viral receptor, ACE2 exhibits a protective role in the cardiovascular system and many other organs and contributes to the absorption of neutral amino acids in intestine (Kuba et al., 2010).

The ACE2 gene and protein show a high degree of genetic polymorphisms including single nucleotide variation, transcriptional variation, post-transcriptional modifications and putative protein mutations (Devaux et al., 2020a), among which single nucleotide polymorphisms (SNPs) has made its way to the scientific spotlight. Recently, Suryamohan et al. (2021) found 298 unique protein-altering variants across 256 codons distributed throughout the 805 amino acid long human ACE2 (hACE2) from a number of databases (Suryamohan et al., 2021). It is noteworthy that the symptoms and severity of COVID-19 vary greatly, ranging from very mild or no symptoms to pneumonia, acute respiratory distress syndrome (ARDS), and even death (Lopera Maya et al., 2020). As SARS-CoV-2 primarily depends on ACE2 for fusion and entry, ACE2 variation is considered to be one of the causes for this difference. Thus, it’s important to systematically characterize and evaluate ACE2 polymorphism. Herein, this review provides an update on the possible role of ACE2 variants in both the susceptibility of people to SARS-CoV-2 infection and the outcome of COVID-19. Meanwhile, particular attention is given to ACE2-based potential therapeutic strategies.

## Altered Affinity of ACE2 Variants for S Protein

Considering that close and stable contact is crucial for RBD recognition, residue changes of ACE2 in the binding interface would influence affinity that is one of the most important determinants of host susceptibility. K353 and K31 in hACE2, which have been shown to be the main hotspots that form hydrogen bonds with the main chain of N501 and Q493 in receptor-binding motif respectively, play a role in tightly binding to the S protein of SARS-CoV-2 (Sehailia and Chemat, 2020). Due to possessing identical or similar critical residues, ACE2 molecules from pigs, ferrets, cats, orangutans and monkeys could bind to SARS-CoV-2 RBD with analogous efficiency (Wan et al., 2020). However, the presence of N30 (instead of D30) and N31 (instead of K31) in mouse ACE2 would cause the lack of salt bridges and critical H-bond in the ACE2-SARS-COV-2 RBD complex. In terms of rats, although E26 (instead of K26) in the ACE2 forms a salt bridge with K417 in RBD, it drives the relative movement of RBD over the rat ACE2 (a shift of 6.5 Å comparing to its relative position in the human complex) and contributes to a decrease in the affinity of the virus to the ACE2 receptor (Brooke and Prischi, 2020). Another hotspot residue, K353, was replaced by histidine in mice and rats, leading to unfavorable bind with the SARS-CoV-2 RBD (Gao and Zhang, 2020). Thus, ACE2 receptor polymorphism can predict species susceptibility to SARS-CoV-2 to some extent (Devaux et al., 2020b). Although pigs and ferrets can be infected with SARS-CoV-2, they generally have a large size and a long growth cycle that is difficult to process in large quantities. Given the rapid emergence of virus mutations and novel vaccine candidates, there is a continuous need for genetic-modified mouse/rat models with the high tissue-specificity of hACE2 expression and susceptibility to SARS-CoV-2 infection.

Even if a total of more than 4 billion vaccine doses have been administered at present, COVID-19 is still spreading rapidly around the world with risk factors for infection including race/ethnicity, ages and both sexes. Thus, it is particularly important to pay close attention to the genetic variation of hACE2. The majority of the ACE2 coding variants found in Genome Aggregation Database (gnomAD) are missense variants that can alter amino acid sequence of the encoded protein, and ultimately result in a change of protein structure. Ser19, situated at the beginning of the helix S19-I54, forms hydrogen bonds with Glu23 and Gln24 to stabilize the helical structure and may interact with the S477 in S protein *via* weak hydrophilic interaction. Its mutation to proline would destabilize the helix structure and consequently influence the recognition by SARS-CoV-2 (Guo et al., 2020). Based on the OncoMX (https://oncomx.org/) and gnomAD (https://gnomad.broadinstitute.org/) datasets, we compared 23 ACE2 variants with different allele frequencies in European (non-Finnish) and East Asian (**Table 1**). It is shown that 14 ACE2 variants (I21V, E23K, K26R, N64K, T92I, Q102P, D206G, G211R, R219C, E329G, H378R, V447F, A501T and N720D) with enhanced susceptibility have higher allele frequencies in European (non-Finnish) populations than East Asian populations, and 2 ACE2 variants (E35K and F72V) which offer resistance have higher allele frequencies in East Asian populations while they are low or not expressed in European (non-Finnish) populations. These findings are consistent with the epidemic situation and could partially account for the differences between COVID-19 prevalence and mortality rates in Europe and East Asia.

**Table 1:** Allele frequencies of ACE2 variants with altered affinity in European (non-Finnish) and East Asian.

Variant	Wild aa	Position	Mutant aa	Effect on affinity	Direct or indirect	European (non-Finnish)	East Asian
rs778030746	I	21	V	↑	Indirect	0.00002446	0.000
rs756231991	E	23	K	↑	Indirect	0.00001222	0.000
rs4646116	K	26	R	↑	Direct	0.005868	0.00006738
rs1348114695	E	35	K	↓	Direct	0.00001221	0.0001444
rs1192192618	Y	50	F	↓	Indirect	0.00001227	0.000
rs1569243690	N	51	S	↓	Indirect	0.00001229	0.000
rs1325542104	M	62	V	↓	Indirect	0.00001266	0.000
rs1199100713	N	64	K	↑	–	0.00001081	0.000
rs1256007252	F	72	V	↓	Indirect	0.000	0.00007243
rs763395248	T	92	I	↑	Direct	0.00002448	0.000
rs1395878099	Q	102	P	↑	–	0.00002168	0.000
rs142443432	D	206	G	↑	–	0.0006314	0.000
rs148771870	G	211	R	↑	–	0.001944	0.000
rs372272603	R	219	C	↑	–	0.0006950	0.000
rs143936283	E	329	G	↑	–	0.00006510	0.000
rs370610075	G	352	V	↓	Direct	0.00001274	0.000
rs961360700	D	355	N	↓	Indirect	0.00002591	0.000
rs142984500	H	378	R	↑	–	0.0001950	0.000
rs762890235	P	389	H	↓	–	0.00002453	0.000
rs776328956	V	447	F	↑	–	0.00007882	0.000
rs191860450	I	468	V	↑	–	0.00002198	0.01140
rs140473595	A	501	T	↑	–	0.00004746	0.000
rs41303171	N	720	D	↑	–	0.02521	0.000

Notably, there are still controversial issues in results of binding affinity of S protein for the same ACE2 receptor among studies. Suryamohan et al. (2021) and Wang et al. (2020) predicted that S19P, T27A and M82I enhanced ACE2 affinity whereas E37K, G326E, G352V and D355N exhibited decreased binding to RBD of S protein (Wang et al., 2020; Suryamohan et al., 2021). In contradiction to their results, another group has reported that all these variants were found to have no obvious changes in the binding energy scores (Othman et al., 2020). These discrepancies may be attributed to different criteria and methods used by various researchers. Utilizing 5 fast structure-based computational approaches, Hadi-Alijanvand and Rouhani (2020) reported the predicted affinities of 240 mutated versions of ACE2 with the SARS-CoV-2 Spike protein, and there were varying degrees of difference in these prediction results. Particularly, the predicted stabilities of 211 ACE2 variant/S1 complexes obtained using SEPAS and SAAMBE-3D methods were different, among which 101 were completely opposite (Hadi-Alijanvand and Rouhani, 2020). As these methods considered different structural aspects of protein complexes, the observed inconsistency in prediction seems natural. Therefore, to better identify the risk variant in ACE2 and develop genetic diagnostic risk profiling for COVID-19 susceptibility, it is essential not only to develop more accurate methods that consider the dynamic features of protein structures, but also to perform more validation studies *in vitro*.

## Sex and Age Differences in ACE2 Polymorphisms

In the early pandemic, it was generally believed morbidity and lethality were higher in men compared with women. Although the available data indicate similar SARS-CoV-2 infection rates between the genders, males account for a greater percentage of deaths in most countries throughout the world, including China (approximately 64% male: 36% female), France (57.83%: 42.17%), Italy (56.43%: 43.57%), America (54.85%: 45.15%) and England (54.44%: 45.56%) (Global Health 50/50). As is well known, genetic variants of ACE, angiotensinogen and angiotensin II (Ang II) receptor genes show more profound effects in men, some of which are only positive in them. Therefore, apart from a higher prevalence of smoking and a deadly symbiosis of chronic systemic inflammation produced by chronic metabolic disease (such as obesity, metabolic syndrome and type 2 diabetes) in males (Mauvais-Jarvis, 2020) and a stronger innate and adaptive immune response to contagions in females, it is conceivable that ACE2 and its genetic variants also display gender-specific effects, which might be more decisive than epigenetic regulation for SARS-CoV-2 progression. For most X-chromosome genes, the dosage difference between XX females and XY males is balanced by silencing one of the two X chromosomes in females in early development. However, the ACE2 gene is located on Xp22.2, an area where genes could escape this X-inactivation, resulting in phenotypic differences between sexes. In males, the 16 residues of RBD carried by SARS-CoV-2, are in contact with 16 out of 20 residues of ACE2 protein (Lan et al., 2020). In females, the same SARS-CoV-2 RBD can be recognized by ACE2 on either of the two X chromosomes while cannot perfectly bind to the same residue sequences of ACE2 on the second chromosome theoretically, allowing unbound ACE2 to catalyze the cleavage of Ang II to form Ang-(1-7), and thus reducing unregulated inflammatory lung damage. With a single X chromosome, men lack the alternative mechanisms of organ protective functions. In addition, males are hemizygous for the ACE2 gene, but in females, the rate of homozygous for variants was extremely low and 98.3% of missense variants were heterozygous, implicating that the efficacy of interaction-booster variants of ACE2 can be stronger in men than women. An analysis revealed that 7 of 10 interaction-booster variants (S19P, K26R, I21V, I21T, N64K, H378R, and T92I) were expressed in males, which means more than half of the variants have the opportunity to affect the interaction between the hACE2 and S1 protein of SARS-COV-2 (Darbani, 2020). Nevertheless, the current data is not able to determine a clear causality, and as most of the identified variants have very low frequencies, further functional studies are needed to verify these results.

The high incidence of COVID-19 in the elderly is also associated with increased expression of ACE2 (Pruimboom, 2020). In children, the ACE2 gene in the lungs, oral tissues, and other organs is ordinarily hypermethylated and therefore actually silent (Pruimboom, 2020). Nevertheless, high-throughput, next-generation sequencing technologies and microarray revealed an increase in variability of DNA methylation with age, which caused certain genes to gradually become more active during the aging process. For example, DNA methylation at one CpG (cg08559914) near the transcription start site of the ACE2 gene in airway epithelial cells is inversely correlated with biological age (Corley and Ndhlovu, 2020). In addition, a previous study showed that ACE2 expression is also transcriptionally regulated by adenosine monophosphate kinase *via* sirtuin 1 (SIRT1) (Patel et al., 2016), and exogenous zinc strongly inhibits SIRT1 activity (Chen et al., 2010), hence, regulation of SIRT1 by zinc may decrease ACE2 expression and ultimately SARS-CoV-2 entry into the cell. However, some evidence indicates that zinc intakes among older adults might be marginal. An analysis of the third National Health and Nutrition Examination Survey data found that 35% to 45% of people over 60 years old had zinc intakes below the estimated average (Ervin and Kennedy-Stephenson, 2002). Notably, anosmia and ageusia, previously thought to be pathognomonic of zinc deficiency, are now also considered early signs of COVID-19, suggesting the presence of common mechanisms.

Mortality rates of COVID-19 vary dramatically across age groups. Compared with 18- to 29-year-olds, the rate of death is 95 times higher in 65- to 74-year-olds, 230 times higher in 75- to 84-year-olds, and 600 times higher in those who are 85 years and older (Centers for Disease Control and Prevention, 2021). Apart from chronic conditions and a dysregulated immune system with aging, other possible explanations for this phenomenon are as follows: (1) The vast majority of deaths result from ARDS with the histologic finding of diffuse alveolar damage (DAD). In response to DAD, type II pneumocytes enter the cell cycle and normally downregulate ACE2 expression (Baker et al., 2021). However, in the elderly suffering from COVID-19 or other diseases, the increased circulating inflammatory cytokines and age-associated epigenetic dysregulation preclude this downregulation or even lead to ACE2 upregulation, finally causing worse clinical outcomes. (2) The sACE2 levels are lower in the population over 60 years of age. In addition to the decreased Ang II/ADAM17 induced ACE2 shedding owing to the lower plasma renin concentration; some microRNAs (miRNAs) target the 3’-UTR, 5’-UTR or coding DNA sequence of ACE2 and influence its expression either by mediating complementary mRNA degradation or by interfering with translation. For example, miR-212-5p is significantly positively correlated with age and negatively related to sACE2 while miR-3909, the miRNA significantly negatively correlated with age, is positively related to sACE2 (Elemam et al., 2021). (3) As the active form of vitamin D (VitD), 1,25(OH)2D3 can induce ACE2/Ang-(1-7)/MasR axis activity and suppress renin and the ACE/Ang II/AT1R axis, thereby producing a potential protective role against ARDS. Over 60 years of age, a reduction in the synthesis of VitD in response to UVB becomes apparent, which further increases when getting older (Heaney, 2006).

## Comorbidities and ACE2 Polymorphisms

Although the T allele of ACE2 rs2106809 has been shown to be inversely correlated with severe malaria (De et al., 2021), accumulating evidence suggests that most ACE2 polymorphisms could lead to a variety of diseases, such as atrial fibrillation, hypertension, type 2 diabetes mellitus (T2D), cardiomyopathy and so on. A summary of these results (Lieb et al., 2006; Yi et al., 2006; Wang et al., 2008; Meng et al., 2015; He et al., 2018; Kumar et al., 2018; Liu C. et al., 2018; Pan et al., 2018; Luo et al., 2019; Lozano-Gonzalez et al., 2020; Liu et al., 2021) and related research is shown in **Table 2**. Notably, ethnicity has been proved to alter the link between ACE2 single nucleotide polymorphisms (SNPs) and some diseases. For example, among persons of British (rs1978124, rs2074192 and rs4646188) or Finnish (rs879922) descent, there is no correlation between ACE2 SNPs and T2D, however, all the four SNPs are dramatically relevant to T2D in Xinjiang Uygur. Similarly, the sex dimorphism is observed in the relationship between ACE2 SNPs and certain diseases. The left ventricular mass index of males with the minor alleles of three SNPs (rs879922, rs4240157 and rs233575) is higher than those who do not carry these SNPs, but this phenomenon is not observed in women. On the contrary, the association of ACE2 rs2106809 with blood pressure response to ACE inhibitor was identified only in women but not in men. Specifically, after 6 weeks of ACE inhibitor therapy, female patients carrying CC or CT genotype had a greater degradation of diastolic blood pressure compared with those carrying TT genotype (Chen Y. Y. et al., 2016). Therefore, it is reasonable to assume ACE2 variant rs2106809 T allele or some other SNPs in the same linkage disequilibrium block, may be functional and could decrease the sACE2 expression and increase the susceptibility to hypertension. Considering the double effect of sACE2 on hypertension and antiviral activity, this may partly explain the high incidence rate of hypertension (58%) in critically ill patients with COVID-19 (Sulaiman et al., 2021). By the way, ACE2 rs2074192 T allele and rs2285666 G allele, which are listed in the **Table 2**, have been reported to be associated with more severe clinical outcomes of SARS-CoV-2 infection (Cafiero et al., 2021; Mohlendick et al., 2021). Thus, besides factors such as aging, vascular disorders and delayed virus clearance, variants-mediated risks are worth exploring in more detail. Additionally, lupus, diabetes and cancer are also comorbidities closely related to adverse outcomes or death. It is easy to understand this phenomenon because aberrant epigenetic modification of ACE2 gene is one of the characteristics of these diseases. DNA methylation defects in lupus patients (Sawalha et al., 2020), aggravated by oxidative stress generated from SARS-CoV-2 infection, could lead to ACE2 overexpression and enhance viral entry. Therefore, therapeutic strategies and drugs that efficiently target epigenetic modification should be considered in further research.

**Table 2:** Association between ACE2 polymorphisms and human diseases/ symptoms in different populations.

Disease/Symptom		Subjects’ characteristic	Variant																Reference(s)
			rs2285666	rs4240157	rs2074192	rs4646188	rs879922	rs2048683	rs233575	rs4646156	rs1978124	rs714205	rs2106809	rs6632677	rs4830542	rs2236306	rs4646142	rs4646155	
Cardiac event	Higher risk of AF	EH subjects from the south Xinjiang	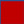	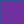											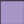		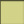	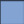	Luo et al., 2019
	Higher risk of AF	Uygur T2D patients		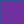	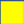	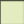													Liu et al., 2021
	Larger size of LAD	Uygur T2D patients		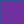	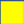	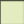													Liu et al., 2021
	Lower LVEF	Uygur T2D patients		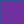		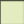				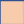									Liu C et al., 2018
	Higher parameters (LVMI and SWT) of LVH	Uygur T2D patients		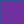		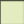	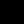	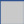		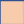									Liu C et al., 2018
	Higher parameters (LVMI and SWT) of LVH	German men from the Augsburg area		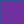			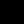		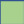	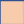									Lieb et al., 2006
	Higher parameters (LVMI and SWT) of LVH	Male HCM patients											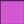	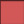					Wang et al., 2008
	DCM	Donors from North Indian ethnicity												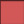					Kumar et al., 2018
Blood pressure disorder	EH	Subjects from the south Xinjiang		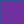	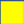	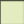	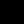						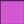		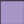			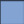	Pan et al., 2018; Luo et al., 2019
	EH	Dongxiang population	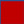																Yi et al., 2006
	Higher levels of SBP	Donors from Mexico City and surrounding states		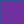				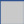											Lozano-Gonzalez et al., 2020
	Higher levels of SBP	Uighurs		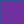		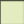	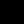	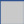	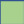	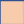									Liu C et al., 2018
	Higher levels of DBP	Donors from Mexico City and surrounding states		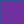				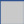											Lozano-Gonzalez et al., 2020
	Higher levels of DBP	Uighurs			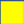	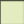	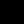												Liu et al., 2018
Dyslipidemia	High LDL-C level	Uygur T2D patients					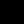	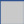		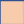									Liu et al., 2018
	High LDL-C level	Subjects from the south Xinjiang				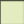	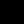		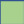		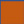					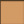			Pan et al., 2018
	High CHOL level	Uygur T2D patients					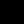	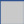		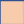	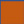								Liu C et al., 2018
	High CHOL level	Subjects from the south Xinjiang		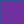	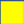	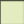	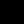			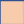					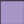				Pan et al., 2018
	High TRIG level	Uygur T2D patients		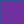			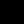												Liu C et al., 2018
	High TRIG level	Subjects from the south Xinjiang				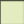							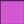						Pan et al., 2018
	Low HDL-C level	Uygur T2D patients		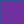	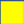		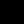	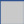	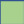	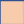									Liu C et al., 2018
	Low HDL-C level	Subjects from the south Xinjiang	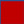			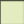							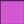				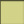	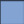	Pan et al., 2018
Ischemic stroke		Subjects from the south Xinjiang		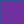		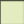									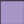				Pan et al., 2018
Increased serum sodium level		Uygur T2D patients		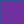		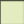													Liu et al., 2021
Lower serum potassium concentration		Uygur T2D patients			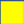	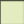													Liu et al., 2021
CAS ≥ 50%		Uygur T2D patients			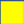		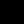												Liu C et al., 2018
CAS ≥ 50%		EH subjects from the south Xinjiang	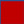		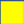												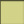		Luo et al., 2019
CAS ≥ 50%		Uighurs			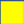		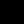												Liu C et al., 2018
T2D		Uighurs		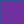	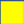	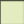	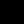	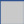	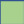	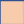	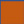								Liu C et al., 2018
Increased MAU level		Uygur T2D patients		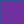	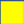	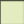	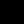												Liu C et al., 2018
Higher level of HsCRP		Uygur T2D patients		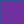		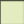													Liu et al., 2021
DR and PDR		Chinese female T2D patients			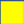							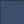							Meng et al., 2015
SGA		Neonates			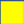														He et al., 2018

13 ACE2 variants are shown in different colors and have been associated with different diseases or symptoms.

AF, atrial fibrillation; LAD, left atrial end−systolic diameter; LVEF, left ventricular ejection fraction; LVMI, left ventricular mass index; SWT, septal wall thickness; LVH, left ventricular hypertrophy; DCM, dilated cardiomyopathies; EH, essential hypertension; SBP, systolic blood pressure; DBP, diastolic blood pressure; LDL-C, low-density lipoprotein cholesterol; CHOL, cholesterol; TRIG, triglyceride; HDL-C, high-density lipoprotein cholesterol; CAS, carotid arteriosclerosis stenosis; T2D, type 2 diabetes; MAU, microalbuminuria; HsCRP, high-sensitivity C-reactive protein; DR, diabetic retinopathy; PDR, proliferative diabetic retinopathy; SGA, small-for-gestational-age; HCM, hypertrophic cardiomyopathy.

## ACE2-Based Pharmacotherapy for COVID-19

Up to date, SARS-CoV-2 variant B.1.617 (Delta) has spread voraciously across more than 100 countries and its major mutation site, L452R, enhances infectivity by increasing the affinity of the S protein to the hACE2 (Deshpande et al., 2021). Tremendous advances have been made in vaccine development and nonpharmaceutical interventions to stop the spread of COVID-19 infection, but treatments to halt the progression of the disease are still limited. As a competitive interceptor of SARS-CoV-2 and other coronaviruses, sACE2 can be used as a protective biomarker for rapid test screening, and even one of the treatment strategies. By the way, they are not affected by common escape mutations in viral proteins. However, in view of its low concentration in plasma, it’s necessary to introduce exogenous forms. In *in-vitro* cell-culture experiments and engineered human organoids, an appropriate introduction of human recombinant soluble ACE2 (hrsACE2) reduced the SARS-CoV-2 growth by a factor of 1,000–5,000 (Monteil et al., 2020), directly demonstrating that this can significantly neutralize viruses. At the same time, no drug-related severe adverse events were observed in the tests of healthy volunteers and COVID-19 patients (Haschke et al., 2013). Interestingly, Khan et al. (2017) found that the infusion of hrsACE2 tends to decrease the interleukin-6 (IL-6) concentration in patients with ARDS (Khan et al., 2017). Given that the level of IL-6 has a positive correlation with the severity of the disease, hrsACE2 is considered as a promising treatment option. However, the therapeutic potential of recombinant ACE2 (rACE2) for chronic use is restricted due to its short plasma half-life of only 1.8 hours. To circumvent this, rACE2-Fc is created by fusing rACE2 and an Fc fragment, with a plasma half-life of 174.2 hours and higher serum activity than rACE2 (Liu P. et al., 2018). Similarly, a C-terminally truncated hrsACE2 protein of 618 amino acids is fused with a small (5-kD) albumin-binding domain (ABD) to form a new type termed ACE2 1–618-ABD (Wysocki et al., 2021). It offers a better way to prevent viral escape and affords more convenient dosing schedules compared with the native form of sACE2 1–740 currently undergoing clinical trials (NCT04335136). In addition, after viral binding, ACE2-ABD could direct the SARS-CoV-2–ACE2 complex to a different cell sorting pathway. When bound to albumin, it might direct the complex into the FcRn-mediated recycling pathway rather than the lysosome-endosome pathway that seems crucial for viral processing and replication.

Besides rACE2, ACE2-derived peptides corresponding to the ACE2-interacting domain of SARS-CoV-2 may represent an alternative strategy to target this pathway due to their relative safety, low production complexity and remarkable curative action. Through fusing the RBD-binding proteins to the CHIPΔTPR modified E3 ubiquitin ligase domain, Chatterjee et al. (2020) designed 23-mer (A2N)-CHIPΔTPR fusion, and this ACE2-derived peptide demonstrated robust RBD degradation capabilities in human cells and reduced the infection rate of the pseudovirus by ~60% (Chatterjee et al., 2020). As for genuine SARS-CoV-2, its infection has also been reported to be inhibited by Spike-targeting ACE2-derived peptide1 (SAP1) and SAP6 in affinity precipitation assays (Larue et al., 2021).

Of note, although most ACE2-based pharmacotherapies exhibit an acceptable safety profile and some of them have entered clinical trials (**Table 3**) (Zhong et al., 2011; Zou et al., 2014; Chen L. J. et al., 2016; Gu et al., 2016; Khan et al., 2017; Liu C. et al., 2018; Rathinasabapathy et al., 2018; Chatterjee et al., 2020; Clinicaltrials, 2021; Larue et al., 2021; Wysocki et al., 2021), the understanding of SARS-CoV-2 pathogenesis is still in a developing stage. The possibility of virus making use of other receptors (e.g., tyrosine-protein kinase receptor UFO, kidney injury molecule-1 and cluster of differentiation 147), co-receptors/auxiliary proteins or even other mechanisms for entry into the cells cannot be ruled out. As the clinical manifestations of SARS-CoV-2 infection are very variable and can occur from asymptomatic infection to severe pneumonia, the appropriate drug concentrations in different patients should be explored in future research. Moreover, only the effect of hrsACE2 on early SARS-COV-2 infection is understood until now, its effect in the later stages of the disease process is still unclear, and the effects on the lungs are also poorly understood. ACE2 infusion may increase Ang-(1-7) levels and reduce circulating Ang II levels to promote infectious or cardiogenic effects in later stages of the disease. Thus, the concentration in the same patient at different stages of the treatment must be precisely adjusted to remain within the therapeutic range. Furthermore, the human body’s immune response to recombinant human proteins may result in the development of neutralizing antibodies and specific T cell responses, thereby compromising therapeutic potential. Therefore, before these types of peptides are approved for the clinical treatment of COVID-19, sufficient data from *in vitro* and *in vivo* studies are still needed to confirm their efficacy. Additionally, except for intravenous treatment, nasal drug administration should be paid more attention because of its fast onset of action, increased bioavailability and reduced adverse reactions.

**Table 3:** A summary of the therapies based on ACE2 used until present.

Primary drug		Condition or disease	Species	Dosage	Medication method	Results	Pros and Cons	Reference
rACE2	hrsACE2	Pulmonary hypertension	Mice (FVB/N)	1.2 mg/kg/day × 14 days	Mini-osmotic pumps	Significantly attenuated vascular remodeling and increased pulmonary SOD2 expression without measurable effects on pulmonary fibrosis.	–	Rathinasabapathy et al., 2018
	hrsACE2	COVID-19	Human	2 times/day	Intravenous injection	Significant improvement in mechanical ventilator-free Days and reduction in viral RNA load observed	–	Clinicaltrials, 2021
	hrsACE2	H5N1 infection induced acute lung injury	Mice (BALB/c)	0.1 mg/kg × 3 times	Intraperitoneal injection	Reduced acute lung injury severity and prolonged overall survival time of infected mice	–	Zou et al., 2014
	hrsACE2	ARDS	Human	0.4 mg/kg × 2 times/day ×3 days	Intravenous injection	Did not result in improvement in physiological or clinical measures of ARDS	Causes some adverse events, such as hypernatremia, pneumonia, dysphagia and rash.	Khan et al., 2017
	hrsACE2	Respiratory syncytial virus induced lung injury	Mice (C57BL/6 )	0.1 mg/kg × 3 times	Intravenous injection	Reduces the severity of lung injury.	–	Gu et al., 2016
	hrsACE2	Ang II- mediated renal oxidative stress, inflammation, and fibrosis	Mice (C57BL/6)	2 mg/kg/day × 14 days	Intraperitoneal injection	Reversed renal NADPH oxidase activation and proinflammatory changes, and attenuated tubulointerstitial fibrosis	–	Zhong et al., 2011
	hrsACE2	Ang II-induced renal fibrosis	Mice (apoE-KO)	2 mg/kg/day × 14 days	Intraperitoneal injection	Dramatically ameliorated Ang II-mediated hypertension, kidney remodeling and tubulointerstitial fibrosis	–	Chen et al., 2016a
	rACE2-Fc	Acute hypertension	Mice (BALB/c)	4 kU/kg	Intravenous injection	Significantly lowered SBP following Ang II	1. With the same dose, the peak blood concentration of rACE2-Fc was 2-fold higher than that of untagged rACE2 and could be sustained for much longer time. 2. Infusion of rACE2-Fc did not show any blood pressure-lowering effect in normotensive controls. 3. It can pass through the placental barrier and is expected to contribute to Ang II degradation during the placental passage and in the fetus. 4. Long-acting rACE2-Fc may help to address the “Ang II escape” phenomena.	Liu P et al., 2018
	rACE2-Fc	Chronic hypertension, albuminuria and multiorgan fibrosis	Mice (RenTgMK)	1 time/7 days × 42 days	Intravenous injection	1. Reduced endogenous plasma Ang II levels 2. The levels of BP and albuminuria stayed within normal ranges 3. Reduced areas of fibrosis 4. Reduced the levels of Akt and Erk phosphorylation levels		Liu P et al., 2018
	rACE2-Fc	Cardiac hypertrophy and fibrosis	Mice (C57BL/6)	1 time/7 days × 28 days	Intravenous injection	Completely prevented SBP increase, ventricular wall thickening, heart enlargement, cardiomyocyte diameter increase and interstitial and perivascular cardiac collagen deposition		Liu P et al., 2018
	ACE2 1–618-ABD	SARS-CoV-2 infection	Human kidney organoids	0.2 mg/ml	–	Markedly reduced viral replication	Prolonged duration of action and afforded more convenient dosing schedules.	Wysocki et al., 2021
	ACE2 1–618-ABD	Acute hypertension	Mice (C57BL/6)	1 mg/kg	Intraperitoneal injection	Significantly attenuated Ang II–induced hypertension		
ACE2 derived peptides	23-mer (A2N)- CHIPΔTPR fusion	SARS-CoV-2 Spike-pseudotyped lentivirus infection	HEK293T cells	0.001 mg/ml	–	Reduced the infection rate of the pseudovirus by ~60%	1. Reduces the risk of immunogenicity 2. Can be readily synthesized or be efficiently packaged for delivery in a lipid nanoparticle or adeno-associated virus	Chatterjee et al., 2020
	SAP1, SAP6	SARS-CoV-2 infection	HEK293T-ACE2-GFP cells	3 mmol/L	–	Resulted in ~2-fold reduction in SARS-CoV-2 infection	–	Larue et al., 2021
	SAP1, SAP6	HCoV-NL63 infection	LLC-MK2 cells	3 mmol/L	–	Resulted in ~3-fold reduced HCoV-NL63 titers	–	Larue et al., 2021

ARDS, acute respiratory distress syndrome; NADPH, nicotinamide adenine dinucleotide phosphate; SBP, systolic blood pressure; BP, blood pressure.

## Discussion

**Figure 2:**
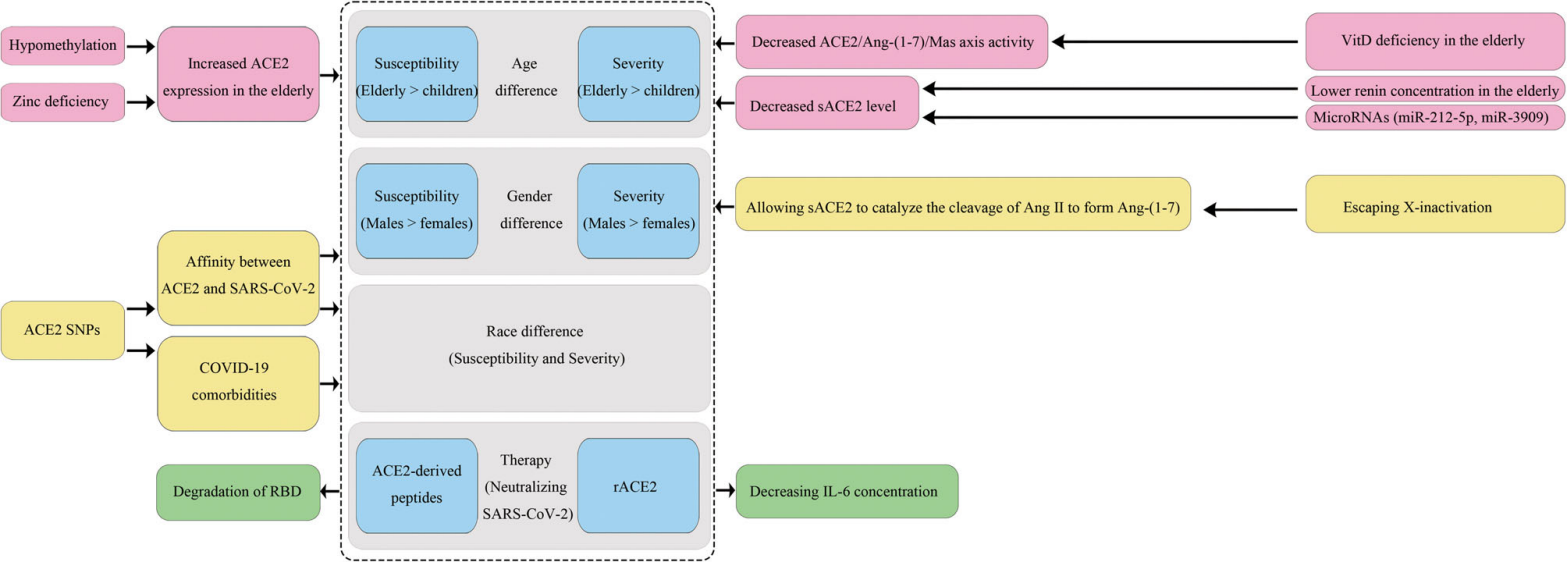
The impact of ACE2 polymorphisms on COVID-19 disease.

In this review, we explored how ACE2 variants and epigenetic factors influence an individual’s susceptibility to SARS-CoV-2 infection and disease outcome in aspects of ethnicity, gender and age, and discussed some ACE2-based COVID-19 treatments (**Figure 2**). Residue changes of ACE2 in the binding interface would influence its expression and affinity with SARS-CoV-2. We compared the allele frequencies and expressions of 23 ACE2 variants in different populations, and found they could partially account for the differences in COVID-19 prevalence and mortality rates. However, in terms of affinity of the ACE2 variant to the S1 protein, current findings are controversial among studies, and the results lack validation by systems biology studies even though some variants have been believed to enhance the affinity in several reports. Thus, there is an urgent need for *in vitro* validation studies to assess the involvements of population-specific SNPs of ACE2 and other host factors in susceptibility toward SARS-CoV-2 infection. Moreover, we believe it is essential to promote the use of multidisciplinary tools and state-of-the-art “omics” technologies to comprehensively define the degree of inter-individual variations in susceptibility and subsequent immune responses determined by gene polymorphisms. Meanwhile, large-scale sequencing projects especially for ACE2 sequencing in the most severe patients in every population should be carried out to identify more specific susceptibility markers.

As an X-chromosome linked phenotype, the interaction-booster variants of ACE2 can have a more substantial impact on males than on females. Moreover, men lack the alternative mechanisms of organ protective functions because they have a single X chromosome, and this would be partially responsible for the gender bias towards a higher mortality rate in males. Furthermore, ethnicity and gender may alter the link between polymorphic variants of the ACE2 gene and a variety of diseases including hypertension, T2D, and cardiomyopathy, which represent risk factors for a severe prognosis of COVID-19. Notably, the high expression of ACE2 gene caused by hypomethylation and zinc deficiency may explain why the elderly are more sensitive to SARS-CoV-2 infection than younger populations and especially children. Moreover, their lower sACE2 and VitD levels cannot protect them from ARDS. Thus, it is reasonable to assume that stimulating specific DNA methylation of ACE2 gene could help to attenuate infection susceptibility and disease severity, and early supplements with zinc and VitD may have special benefits for elderly people. In addition, reducing age-related inflammation may decrease ACE2 expression in type II pneumocytes and limit infection of damaged alveolar epithelium. Indeed, dexamethasone and baricitinib (JAK1/2 inhibitor) have been shown to improve outcomes in COVID-19 patients. Whether these therapies bring benefits, at least in part, by regulating ACE2 expression may be a focus of future research.

Given the emergence of vaccine breakthrough infections, it is still necessary to explore effective treatment strategies and specific antiviral agents against the virus in addition to the development of vaccines. As a competitive interceptor of SARS-CoV-2, exogenous forms of sACE2 can be regarded as promising therapeutic options. Several rACE2 and ACE2-derived peptides have shown acceptable safety and preliminary efficacy, but there are still some challenges to overcome, especially concerning the appropriate drug concentrations in different patients and the same patient at different stages of the treatment.

## Author Contributions

All authors contributed to the literature review for the manuscript. The first draft of the manuscript was written by FC and all authors commented on previous versions of the manuscript. All authors contributed to the article and approved the submitted version.

## Funding

This work was supported by the Natural Science Foundation of Shandong Province (Grant no. ZR2020QC100).

## Conflict of Interest

The authors declare that the research was conducted in the absence of any commercial or financial relationships that could be construed as a potential conflict of interest.

## Publisher’s Note

All claims expressed in this article are solely those of the authors and do not necessarily represent those of their affiliated organizations, or those of the publisher, the editors and the reviewers. Any product that may be evaluated in this article, or claim that may be made by its manufacturer, is not guaranteed or endorsed by the publisher.
